# Fate of mesoangioblasts in a vaginal birth injury model: influence of the route of administration

**DOI:** 10.1038/s41598-018-28967-w

**Published:** 2018-07-13

**Authors:** Marina Gabriela Monteiro Carvalho Mori da Cunha, Giorgia Giacomazzi, Geertje Callewaert, Lucie Hympanova, Francesca Russo, Greetje Vande Velde, Rik Gijsbers, Maarten Albersen, Maurilio Sampaolesi, Jan Deprest

**Affiliations:** 1 Centre for Surgical Technologies, Group Biomedical Sciences, KU Leuven, Leuven, Belgium; 2 Department of Development and Regeneration, Woman and Child, Group Biomedical Sciences, KU Leuven, Leuven, Belgium; 3 Translational Cardiomyology Lab, Stem Cell Biology and Embryology Unit, Department Development and Regeneration, KU Leuven, Leuven, Belgium; 4 Pelvic Floor Unit, University Hospitals KU Leuven, Leuven, Belgium; 5 Institute for the Care of the Mother and Child, Third Faculty of Medicine, Charles University, Prague, Czech Republic; 6 Molecular Small Animal Imaging Center, KU Leuven, 3000 Leuven, Belgium; 7 Laboratory for Molecular Virology and Gene Therapy, KU Leuven, Flanders, Belgium; 8 Department of Urology, University Hospitals Leuven, Leuven, Belgium

## Abstract

Currently cell therapy is considered as an experimental strategy to assist the healing process following simulated vaginal birth injury in rats, boosting the functional and morphologic recovery of pelvic floor muscles and nerves. However, the optimal administration route and dose still need to be determined. Mesangioblasts theoretically have the advantage that they can differentiate in skeletal and smooth muscle. We investigated the fate of mesoangioblasts transduced with luciferase and green fluorescent protein reporter genes (rMABs^eGFP/fLUC^) using bioluminescence, immunofluorescence and RT-PCR in rats undergoing simulated birth injury. rMABs^eGFP/fLUC^ were injected locally, intravenously and intra-arterially (common iliacs and aorta). Intra-arterial delivery resulted in the highest amount of rMABs^eGFP/fLUC^ in the pelvic organs region and in a more homogeneous distribution over all relevant pelvic organs. Sham controls showed that the presence of the injury is important for recruitment of intra-arterially injected rMABs^eGFP/fLUC^. Injection through the aorta or bilaterally in the common iliac arteries resulted in comparable numbers of rMABs^eGFP/fLUC^ in the pelvic organs, yet aortic injection was faster and gave less complications.

## Introduction

Vaginal birth is an important risk factor for the later development of pelvic floor dysfunction^1^. During vaginal delivery, passage of the fetal head results in an excessive and sustained high pressure and deformation of the pelvic floor^2^ leading to both ischemia and reperfusion^3^ and stretch-related injury^4^ to nerves, muscles and supporting structures^5^. Some women do not recover completely after delivery, and may suffer from stress urinary incontinence (SUI), pelvic organ prolapse or fecal incontinence^6^. There are at present no therapeutic interventions that can assist in full recovery of the underlying pathophysiologic vaginal birth-induced changes^5^. Recently, cell therapy has gained attention as a potential treatment for SUI^7,8^. Currently it is considered as an experimental strategy to assist the healing process following simulated vaginal delivery. Several groups have demonstrated functional and morphologic improvements following injection of muscle, adipose and bone marrow derived stem cells (SC) in rodents^9–12^; yet the mechanism of action, the best cell source, the administration route and dose still need to be further explored.

The fate of cells post-implantation is of particular relevance for the therapeutic use of SC since their limited efficacy has been correlated to suboptimal dosing and/or route of administration^13^. It remains unclear whether SC perform best when they incorporate in the tissue at the site of injury/inflammation or whether the effects observed are achieved through paracrine mechanisms. Recent studies have shown that cell therapy was more effective when cells were homing more efficiently or when engraftment was demonstrated^14,15^, supporting the former hypothesis.

Few experimental studies have assessed the biodistribution of cells after local and intravenous administration of SC in animal models for simulated delivery^12,16,17^. Following local injection, the limited diffusion of nutrients and oxygen severely affected the survival of injected cells^18^. Since SC are known to be recruited to sites of inflammation^19^, systemic injection has also been employed. Intravenous administration is the least invasive intravascular route, yet it has the inherent disadvantage that the majority of cells becomes entrapped in the capillary beds of non-target organs, mainly the lungs^20^. An alternative is selective injection, e.g. in the arteries feeding the area of interest which may result in a more efficient engraftment and/or lower cell dose required and less off-target effects^21^. The intra-arterial route has been proven to be effective in many conditions, including renal ischemia-reperfusion injury, radiation injury and skeletal muscle injury^21–23^, however, it has not yet been investigated in the vaginal birth injury model.

In this study, we aimed to investigate the fate of stem cells when administered via local, intravenous or intra-arterial route in a rat model for simulated vaginal birth injury. Here we employ rat-derived mesoangioblasts (rMABs). MABs are vessel-derived SC with a high regenerative potential for muscular disorders^24^. Conceptually, MABs are good candidates for repairing birth injury because of their capacity to differentiate into skeletal and smooth muscle^25^. MABs have already been labeled with a double reporter viral vector encoding both enhance green fluorescent protein (eGFP) and firefly luciferase (fLuc) reporter to monitor their distribution *in vivo* by bioluminescence (BLI) and fluorescence^26^.

## Results

### Characterization of rMABs^eGFP/fLUC^

**Figure 1:**
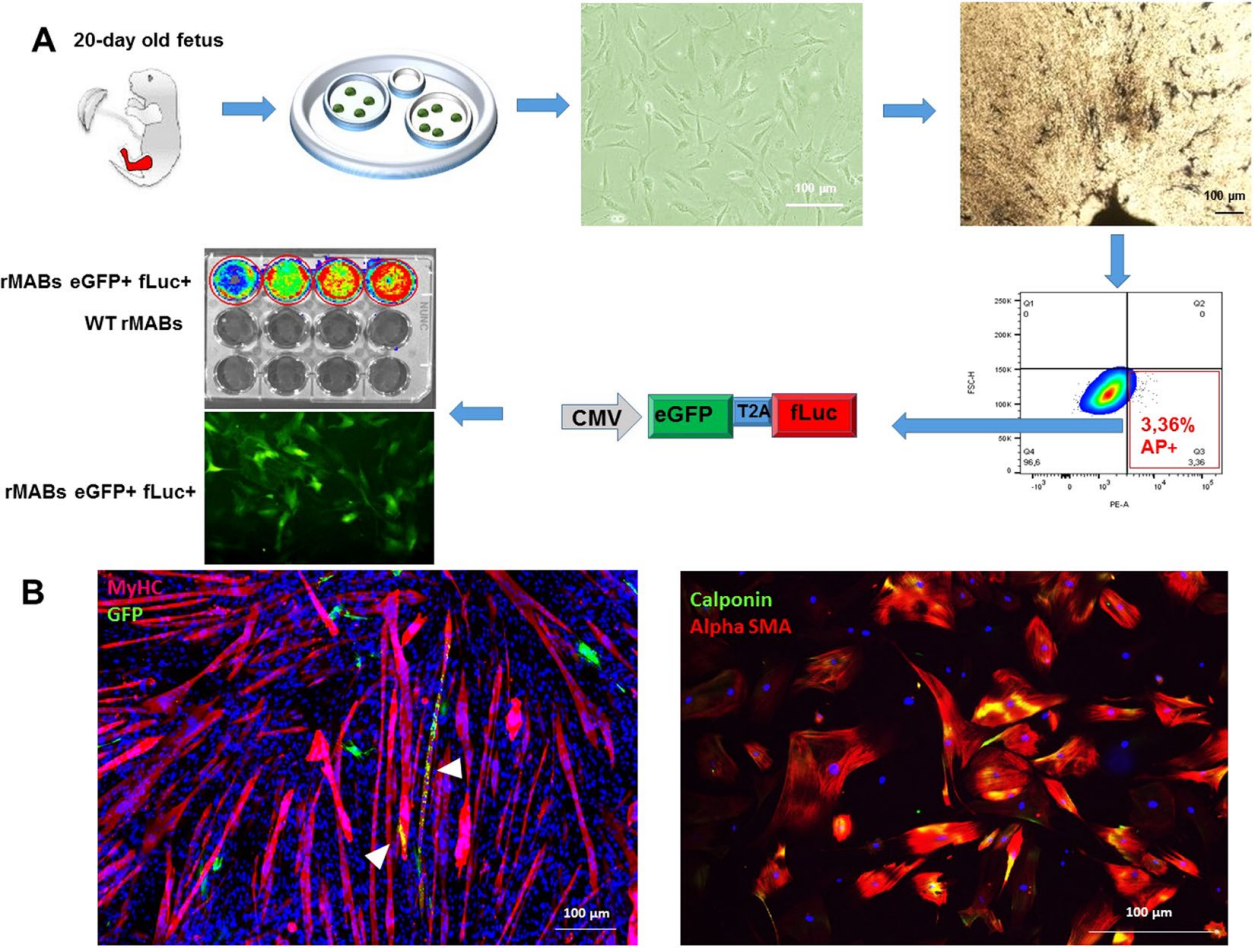
Isolation, manipulation and characterization of rat mesoangioblasts (rMABs). (**A**) rMABs were isolated from 20 days old rat fetuses from skeletal muscle of both hind limbs. After expansion, cells were stained for alkaline phosphatase and further isolated by FACs sorting. In order to track the cells further *in vivo*, rMABs were labelled with CHMWS-eGFP-T2A-fLuc viral vector and sorted by FACs for GFP expression. The engineered cell line of rMABs GPF+ Luc+ were further validated *in vitro* by BLI and immunofluorescence. (**B**) rMABs were differentiated towards skeletal and smooth muscle lineages (left to right). In co-cultures with C2C12 GFP+ rMABs formed few chimeric myotubes (arrow). After smooth muscle differentiation, rMABs expressed both calponin and alpha smooth muscle actin.

Rat MABs were isolated from 20-day-old skeletal muscle fetal biopsies and sorted for alkaline phosphatase after 10 days of culture. About 3% of the total cell population was alkaline phosphatase positive (Fig. 1A). Only fLuc expressing rMABs showed consistent bioluminescence when incubated with luciferin while WT MABs only showed background signal (Fig. 1A).

Next, we investigated the *in vitro* myogenic potential of rMABs^eGFP/fLUC^. To this end, we differentiated them to skeletal and smooth muscle lineages (Fig. 1B). When co-cultured with murine myoblasts (C2C12) they were able to form GFP+ chimeric myotubes, albeit with low efficiency. However, when differentiated towards smooth muscle, rMABs^eGFP/fLUC^ efficiently expressed early and late smooth muscle markers alpha smooth muscle actin and calponin (Fig. 1B). Finally, we profiled rMABs^eGFP/fLUC^ by flow cytometry analysis. rMABs^eGFP/fLUC^ were highly positive for homing and adhesions marker HCAM (CD44) and MCAM (CD146), positive for pericytes marker PDGFR beta (CD140b), and negative for endothelial lineage marker PECAM-1 (CD31) and hematopoietic marker CD45 (Supplementary Figure 1).

### Tracking of carbon particles following local and intra-arterial injections

When Chinese ink was locally injected, black (carbon) particles could be observed on histological sections in the connective tissue around the vagina, rectum and levator ani (Supplementary Figure 2A). There were no carbon particles visible within the interstitial and vascular spaces of these pelvic organs (Supplementary Figure 2C,E,G,I). Conversely, carbon particles were homogeneously distributed in the interstitial and vascular space of pelvic organs when the injection was performed by arterial route (Supplementary Figure 2B,D,F,H,J), indicating that the initial distribution into the target area was effective using these routes of administration.

### Fate of the rMABs^eGFP/fLUC^ after injection through different routes

**Figure 2:**
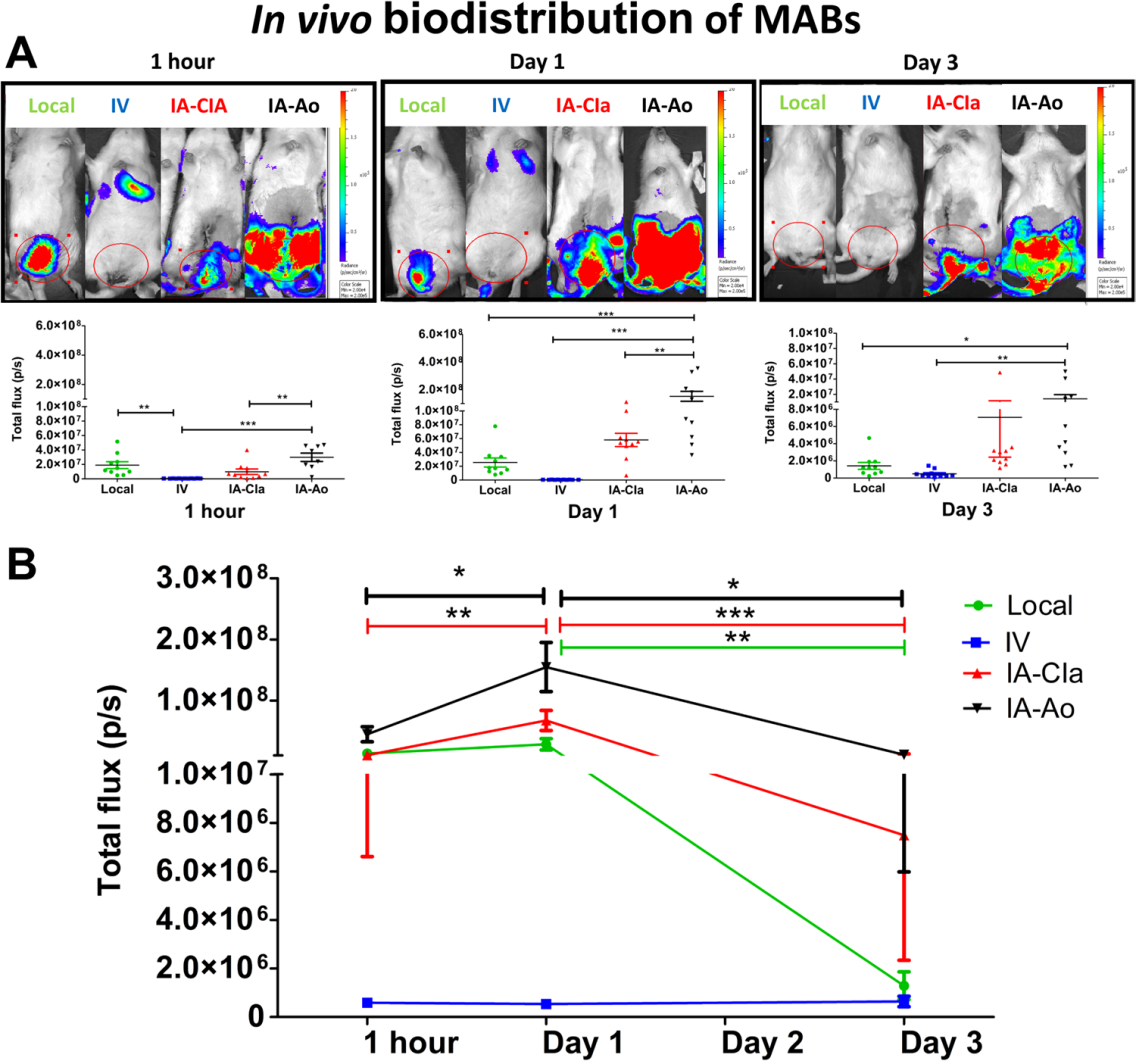
Longitudinal fate of the rMABseGFP/fLUC after local, intravenous and intra-arterial (**A**) Local administration and IA-Ao group showed the highest amount of rMABs^eGFP/fLUC^ in the pelvic organs region 1 h after administration. IA-Ao cohorts showed a significantly higher amount of rMABs^eGFP/fLUC^ at 1 and 3 days compared to local and IV groups. (**B**) Both IA injection groups showed a significant raise of the amount of rMABs^eGFP/fLUC^ at 1d. Local, IA-CIa and IA-Ao groups showed an abrupt drop of rMABs^eGFP/fLUC^ at 3 days. IV- intravenous; IA-CIa- intra-arterial (common iliacs); IA-Ao (intra-arterial (aorta). *p < 0.05; **p < 0.001; ***p < 0.0001.

One hour after injection of rMABs^eGFP/fLUC^, IA-Ao administration route was associated with the highest amount of rMABs^eGFP/fLUC^ in the pelvic organs region, as evidenced by BLI (Fig. 2A, left). Following IA injection, the amount of rMABs^eGFP/fLUC^ increased significantly at 1d (Fig. 2B), with a significantly higher amount of rMABs^eGFP/fLUC^ in the pelvic organs region compared to IV and local administration (Fig. 2A, middle). Following IV injection, most of the BLI signal was found in the lungs and tail at 1 h and 1 day after injection. Animals of all groups showed a significant decrease of rMABs^eGFP/fLUC^ at 3d, yet with the highest remaining cell number in the IA-Ao group (Fig. 2A, right). Individual data are shown in Supplementary Figure 3.

**Figure 3:**
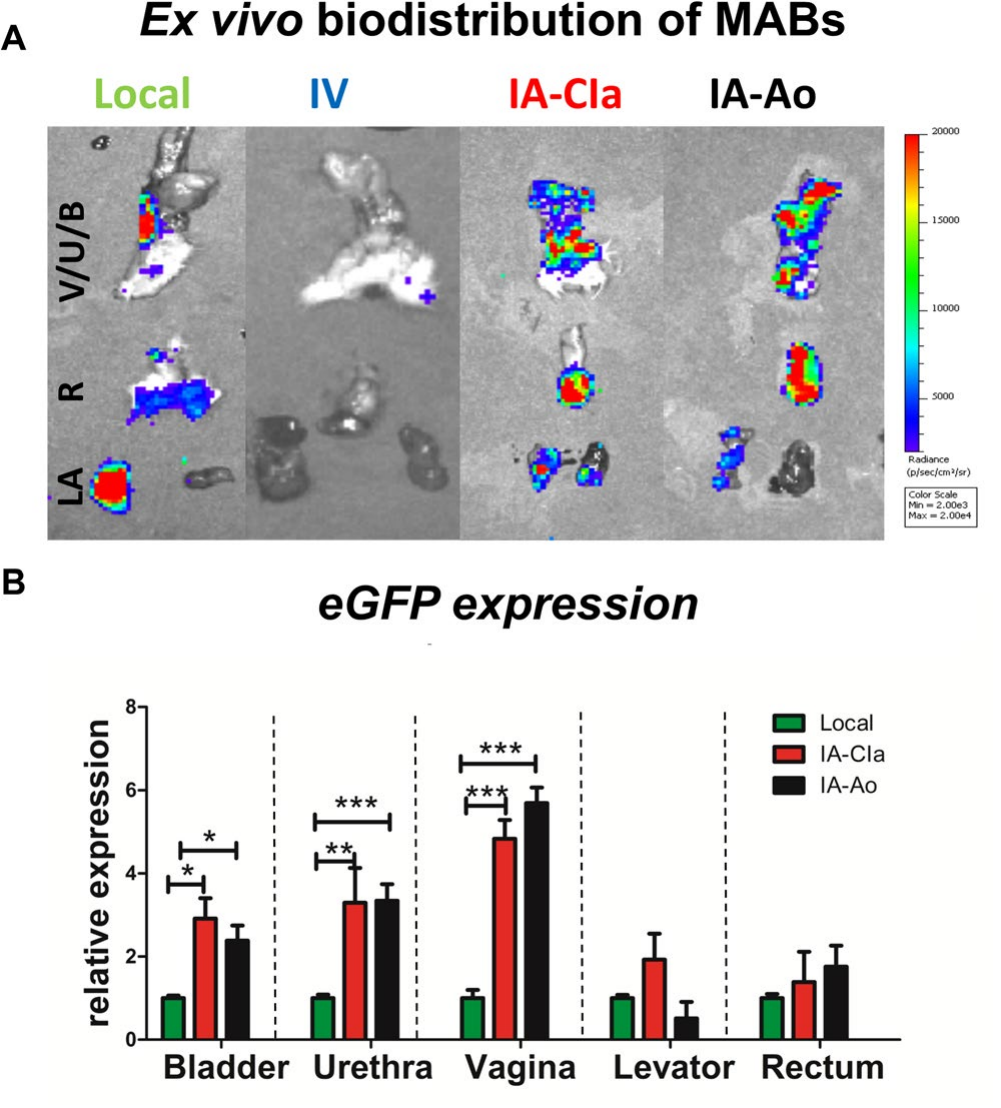
Biodistribution of mesoangioblast (rMABseGFP/fLUC) in the pelvic organs of rats after 3 days of the simulated birth injury. (**A**) *Ex vivo* Bioluminescence analysis of the vagina, urethra, bladder (V/U/B), rectum (R) and levator ani (LA). IA-CIa and IA-Ao showed a more homogeneous distribution of rMABs^eGFP/fLUC^ (**B**) qPCR analysis for GFP expression levels in targeted organs. Data are shown as relative expression, normalized with local injections Mean ± SEM. N = 4, p < 0.05, 2-Way ANOVA, scale bar 100 µm).

**Figure 4:**
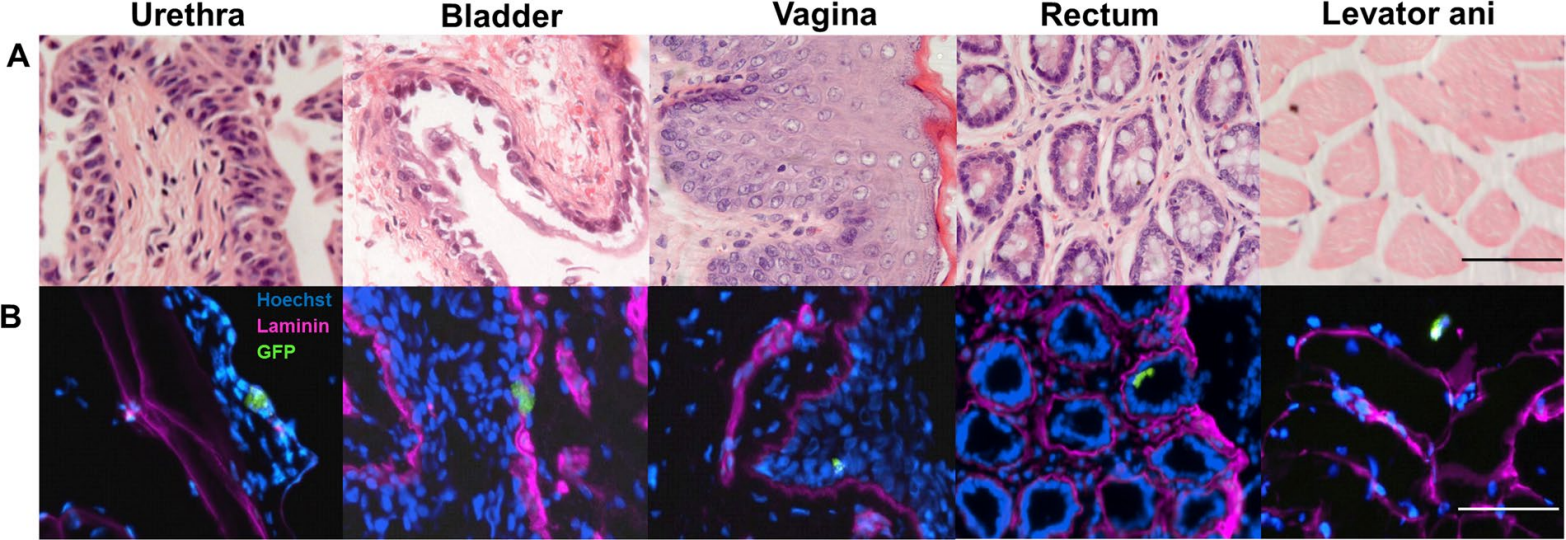
Representative images of rMABseGFP/fLUC administered intra-arterially in simulated birth injury model rats (200x). Green fluorescent protein (GFP) cells could be detected by immunofluorescence in the vagina, bladder, urethra, rectum and levator ani 2 days after injection. Scale bars 50 µm.

When biodistribution was investigated *ex-vivo*, rMABs^eGFP/fLUC^ were more homogeneously distributed in the pelvic organs at 3d following IA administrations (Fig. 3A). A significant drop in the BLI signal intensity was observed at 7 days in all groups. Further, we detected GFP gene expression in the pelvic organs at 3 days and 7 days. Both IA injected animals had a comparable amount of rMABs^eGFP/fLUC^ in the pelvic floor and a significantly higher amount of rMABs^eGFP/fLUC^ in the vagina, urethra and bladder compared to those locally injected at 3 days (Fig. 3B- absolute expression −ΔCT- eGFP local- vagina: −23.112 ± 0.198; rectum: −20.661 ± 0.973; levator ani: −17.460 ± 0.775; urethra: −19.48 ± 0.83; bladder: −20.808 ± 0.94). Again, GFP expression decreased drastically at 7 days in all groups. Further, GFP^+^ cells were tracked in the IA-Ao group by immunofluorescence. GFP^+^MABs could be found in the vagina, bladder, urethra, rectum and levator ani 2 days after injection (Fig. 4).

Sixty percent of IA-CIa rats died due to thromboembolism in the hind limbs. These rats were not included in this study. When we started administrating heparin the rate of thromboembolism dropped to 5% of the rats. Overall, we had a 15% mortality immediately after MABs were injected systemically.

### Influence of the injury on the fate of the rMABs

**Figure 5:**
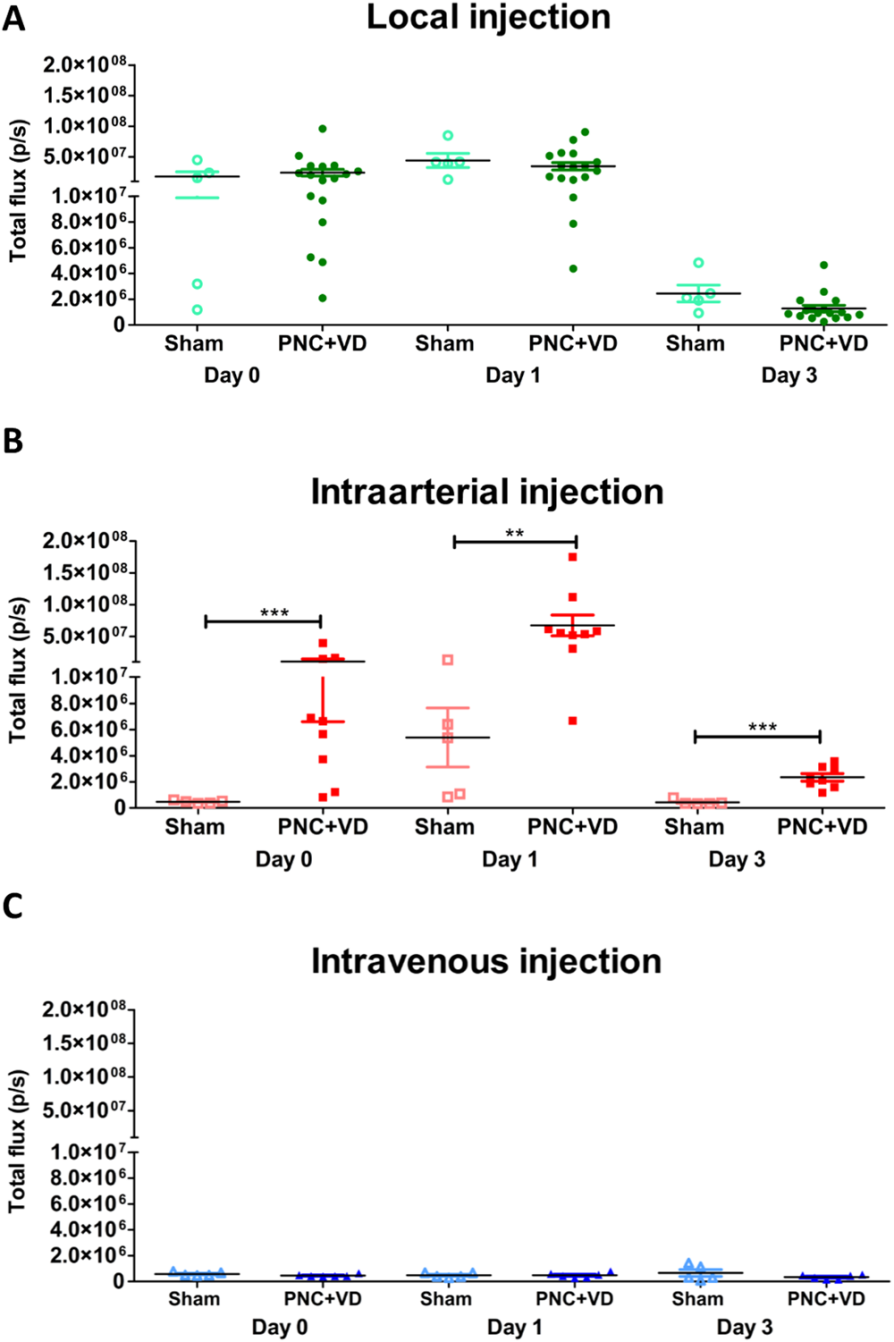
Influence of the injury on the fate of the rMABs A-Presence of rMABs^eGFP/fLUC^ in the pelvic floor are in the non-injured (sham) and simulated birth injury (PNC + VD) model in rats when rMABs^eGFP/fLUC^ were injected locally (**A**), intravenously (**B**) and intra-arterially (**C**). The injury did not affect the fate of the rMABs^eGFP/fLUC^ in the local or IV groups, but in the intra-arterial group. PNC: pudendal nerve crush; VD: vaginal distension. **p < 0.001; ***p < 0.0001.

rMABs were injected in a non-injured group by the IV, local and IA-Cia route in order to investigate the influence of simulated birth injury on the fate of the rMABs^eGFP/fLUC^ (Fig. 5). The injury did not affect the fate of the rMABs^eGFP/fLUC^ in the local or IV groups. However, the non-injured rats showed a significantly lower amount of rMABs^eGFP/fLUC^ compared to the injury cohorts when injected by the intra-arterial route (Fig. 5).

## Discussion

We studied the short-term effect of different routes for rMABs^eGFP/fLUC^ administration in a standardized rat model of birth injury, combining nerve crush and vaginal distension. The most important findings are that intra-arterial delivery resulted in (1) the highest amount of rMABs^eGFP/fLUC^ in the pelvic organs region at 1 and 3 days after injection and in (2) a more homogeneous distribution of rMABs^eGFP/fLUC^ over all relevant pelvic organs early after injection. Moreover, (3) the presence of the simulated birth injury is key for recruitment of intra-arterial injected rMABs^eGFP/fLUC^.

Conceptually yet also clinically, the ideal delivery route is topical so that the transplanted cells can directly and efficiently home to the (therapeutic) target tissues without leakage to other organs, yet avoiding extra damage to the surrounded tissue during the injection. Despite the fact that local injection allowed efficient local delivery of rMABs^eGFP/fLUC ^^12,17^, the cells tended to gather only at the injection sites without being diffused through the pelvic organs. The IV route is usually considered the least invasive technique of injection, however, when injected by this route we could detect relevant numbers of rMABs^eGFP/fLUC^ only in the lungs, as described previously^20^. In light of the limitations of the previous methods for cell delivery in birth injury, we proposed two alternative routes to inject MABs through the aorta and the common iliacs arteries. In this study, we show that rMABs^eGFP/fLUC^ injected intra-arterially resulted in the most efficient homing and homogeneous distribution in the pelvicorgans in the birth injury model. Other researchers have also observed a more homogenous distribution and longer survival of the SC when they were injected IA^23,27^. Moreover, engraftment and regeneration were observed when SC were injected IA in a muscle injury model in a non-primate^28^.

IA-Ao rats showed higher rMABs^eGFP/fLUC^ survival at 1 and 3 days compared to IA-CIa rats. IA-Ao injection had other advantages, such as the need of only one puncture instead of two, leading to a faster procedure with less bleeding and no thromboembolism. Besides, less SC leakage was observed in IA-Ao rats due to technical reasons, given the wider diameter of the aorta compared to the common iliac, which facilitates arterial puncture. Conversely, the occurrence of thromboembolism observed in the IA-CIa group is probably due to the longer compression of the arteries after puncture, required for hemostasis.

It is controversial if the recruitment of the SC is produced by margination, rolling, specific attachment, and extravasation in postcapillaries and venules^29,30^ or whether they are mechanically trapped in capillaries^28^. Our data supports the idea that the recruitment of the rMABs^eGFP/fLUC^ in the IA cohort was due to the inflammatory response since higher amount of rMABs^eGFP/fLUC^ were observed in the injury groups compared to sham controls irrespective of the time point. SC are known for their chemotactic properties due to specifics cytokines, such as monocyte chemotactic protein-3 (MCP-3)^31^. Moreover, MCP-3 is known to play a role in the postischemic recovery and it is upregulated in the pelvic organs immediately after vaginal distension injury^32,33^. One other explanation can be that the inflammatory response increases vasculature permeability, which may lead to higher entrapment of cells in injured organs, similar to the EPR-effect^34^.

We used a vasodilator in order to increase homing of the rMABs^eGFP/fLUC^ injected systemically; as recommended previously^35^. However, we observed an improvement only in the IA group (data not shown). Two possible hypotheses arise: vasodilatation made arterial puncture easier, leading to a lower leakage of rMABs^eGFP/fLUC^; or the vasodilator alleviated the vasoconstriction due to the ischemia and reperfusion injury in the pelvic organs^3,36^, leading to a higher amount of rMABs^eGFP/fLUC^ reaching the target area.

The thromboembolism observed in the IA-CIa group was nearly completely solved by the administration of heparin. We think that the thromboembolism was in part due to the injection of large amount of cells, associated to the arterial injury and the compression after injection, as described previously^37^. Moreover, mesenchymal SC have been shown to have a procoagulant activity inducing thrombogenesis *in vivo*^38^. This effect was avoided by heparin treatment before MSC injection *in vivo*.

Here, we observed a significant increase of rMABs^eGFP/fLUC^ in the pelvic organs region one day after injection, followed by a significant drop at 3 days. Our results are compatible with those of Dai *et al*.^17^, who found an increase of MSC viability at 1 day, followed by a drop after 2 days and completely elimination by 4 days in a birth injury rat model. Interestingly, MSC persisted for 8 weeks in the birth injury BALB nude mice model^12^. Therefore, we hypothesize that this drastic reduction of rMABs^eGFP/fLUC^ viability is due to the adaptive immune system. Currently, cell rejection by the host is a concern in the cell therapy field^26^. Even though, it has been described previously that MABs were inert to the immune-system^39^, isolated T-cells from mMAB-injected muscle were clearly reactive against re-exposure to mMABs^40^. Moreover, it has been proved that the use of immunosuppressors increase the viability of MABs in the long-term^26^. Route of administration and dosage are two critical factors determining the efficiency of cell therapy. Since it has been suggested that a lower dose of SC is necessary for IA injection^21^, the ideal dose for cell therapy in the birth injury model still has to be investigated. Here, we investigated the most efficient homing and biodistribution using several administration routes in the birth injury model. Yet, for investigating their engraftment, it would be wise to use autologous injection or an efficient immunosuppressive therapy^41^. Another limitation of this study is that the functional outcome of the administration routes was not tested. Therefore, a correlation of the fate of the cells in the pelvic area with the effect the MABs may execute has not been established yet. Although most studies have shown that cell therapy was more efficient when administered cells are indeed homing to the target organ^14,15^, further studies will be necessary to elucidate the functional effects of MABs or their secretome in this model.

Altogether, intra-arterial injections of rMABs^eGFP/fLUC^ resulted in a more efficient homing and distribution of mesoangioblasts in the pelvicorgans in rats after birth injury model. Aorta and bilateral common iliac administration resulted in comparable number of MABs in the pelvic organs. From a technical point of view, aortic injection is faster and results in less complication compared to iliac injection.

## Material and Methods

### Generation of reporter rMABs and Cell Culture

In order to investigate the most efficient delivery route for SC in birth injury model, we isolated rMABs from 20-day-old rat fetuses (Fig. 1A). Skeletal muscle from both hind limbs was harvested and processed as previously described^42^. Briefly, tissue biopsies were minced in ~2 mm size pieces and plated on collagen coated 6 cm dishes. After 10 to 14 days alkaline phosphatase positive cells were sorted. rMabs were cultured at 37 °C in a 5% CO_2_, 5% O_2_ humidified incubator in DMEM supplemented with 20% FBS, 1% Pen-Strep, 1% L-glutamine, 1% sodium pyruvate, 1% non-essential amino acids, and 0.2% b-mercaptoethanol, (all reagents from GIBCO,USA). To enable tracking rMABs after injection *in vivo* and in real time, and to prove cell viability, rMABs were transduced with a HIV-derived lentiviral vector LV_CMV-eGFP-T2A-fLuc constructed by the Leuven Viral Vector Core at 1:100 concentration for 48 hours (virus titer 2.34e + 08 TU/ml), and subsequently sorted as GFP^+^ fraction.

### Characterization of rMABs^eGFP/fLUC^

#### Differentiation assays

Differentiation assays were carried out as previously described^42^. Briefly, 2 × 10^4^ rMABs^eGFP/fLUC^ were seeded on 24multi-well collagen-coated plates. Smooth muscle differentiation was induced the day after with DMEM high glucose, supplemented with 2% of heat-inactivated horse serum (HS), 1% penicillin/streptomycin solution, 2 mM glutamine, 1 mM sodium pyruvate, and 10 ng/mL TGFβ (Peprotech, Rocky Hill, USA) for 7 days. Myogenic differentiation was carried out by seeding C2C12 and rMABs^eGFP/fLUC^ in a 1:3 ratio on collagen coated dishes. When cultures reached 80% confluence, myogenic differentiation was induced by incubating the cells with DMEM high glucose, supplemented with 2% of HS, 1% penicillin/streptomycin solution, 2 mM glutamine, and 1 mM sodium pyruvate (all reagents from GIBCO) for 5 days. At the end of differentiations, cultures were fixed with 4% paraformaldehyde (PFA, Polysciences Europe GmbH, Germany) and stained.

### Immunofluorescence staining

Staining was performed following the commonly used steps of Triton-based (Sigma Aldrich, Diegem, Belgium) permeabilization and background blocking with donkey serum (Sigma Aldrich). Cells were incubated overnight with primary antibodies (alpha-Smooth Muscle ActinCy3™; Calponin and anti-GFP) at 4 °C, and after washing, they were further incubated for 1 h with AlexaFluor-conjugated donkey secondary antibodies (Thermo Fisher Scientific, Ghent, Belgium). Nuclei were counterstained with Hoechst. The details of primary antibodies and respective dilutions are described on Table 1.

**Table 1:** List of antibodies used.

Protein	Concentration	Catalog number	Brand	Country
CD44	1/500	ab157107	Abcam	Cambridge, UK
CD140b	1/100	ab32570	Abcam	Cambridge, UK
CD146	1/300	ab75769	Abcam	Cambridge, UK
CD31	1/300	11-0311-85	Thermo Fisher	Gent, Belgium
CD45	1/100	11-0451-82	Thermo Fisher	Gent, Belgium
AlexaFluor 488 goat anti-mouse	1/500	A-11001	Life Technologies	Merelbeke, Belgium
AlexaFluor 647 goat anti-rabbit	1/500	A-11012	Life Technologies	Merelbeke, Belgium
alpha-Smooth Muscle ActinCy3	1/200	C6198	Sigma-Aldrich	Diegem, Belgium
Calponin	1/200	Ab46794	Abcam	Cambridge, UK
GFP	1/400	Ab5450	Abcam	Cambridge, UK
Laminin	1/500	Ab11575	Abcam	Cambridge, UK
Alkaline Phosphatase	1/25	FAB1448P	R&D Systems (Biotechne)	USA

### Flow cytometry analysis

1 × 10^6^ cells were fixed in suspension with 4% PFA for 10 minutes at room temperature. Afterwards cells were equally divided in several polystyrene tubes. Cells were incubated for 30 minutes at 4 °C in the dark with conjugated antibodies against CD44, CD140b, CD146, CD45 and CD31. After two washes in PBS, flow cytometry was performed and data were acquired at the BD FACSCanto™ and analyzed with FlowJo.

### *In Vitro* bioluminescence imaging

rMABs^eGFP/fLUC^ were plated in serial dilution (1.25 × 10^4^–1 × 10^5^) under growth and differentiation medium. When cells reached 80% confluence, 0.3 mg/L D-Luciferin (Promega, Benelux, Leiden, Nederlands) was added to the rMABs^eGFP/fLUC^ and the emitted light photons were detected with the IVIS Spectrum (Caliper Life Sciences, Waltham, Massachusetts, USA). *In vitro* BLI signal intensities were analyzed with Living Image version 4.5 (Caliper Life Sciences).

### Animals and simulated vaginal delivery injury model

The animal experiments were evaluated and approved by the Animal Ethics Committee of the KU Leuven (P271-2015) and was performed according to international guidelines. Sixty-three female virgin Sprague-Dawley rats of 12-week-old (250–300 g) were used. Rats underwent either a simulated childbirth injury by pudendal nerve crush and vaginal distension (PNC + VD; n = 42) or sham (n = 15), as described previously^43^ (Supplementary Figure 4). We chose the combination of pudendal nerve crush and vaginal distention since it mimics better birth injury observed in humans^43^. Rats were anesthetized by intraperitoneal injection of ketamine (70 mg/kg), xylazine (7.5 mg/kg) and buprenorphine (0.05 mg/kg). To induce PNC injury, an incision was made in the dorsolumbar area; the pudendal nerve was identified in the ischiorectal fossa and crushed twice with a needle holder for 30 sec. For simulated VD, a modified 10Fr Foley catheter was inserted into the vagina and the balloon was inflated to 3 mL for 4 h. Sham operations consisted of pudendal nerve dissection without crushing and catheter insertion for 4 hours without balloon inflation. All animals were kept on a heating pad during surgery and recovery. For post-operative pain-relief, buprenorphine was administered IP for 2 days (0.1 mg/kg, BID).

### Injection routes

**Figure 6:**
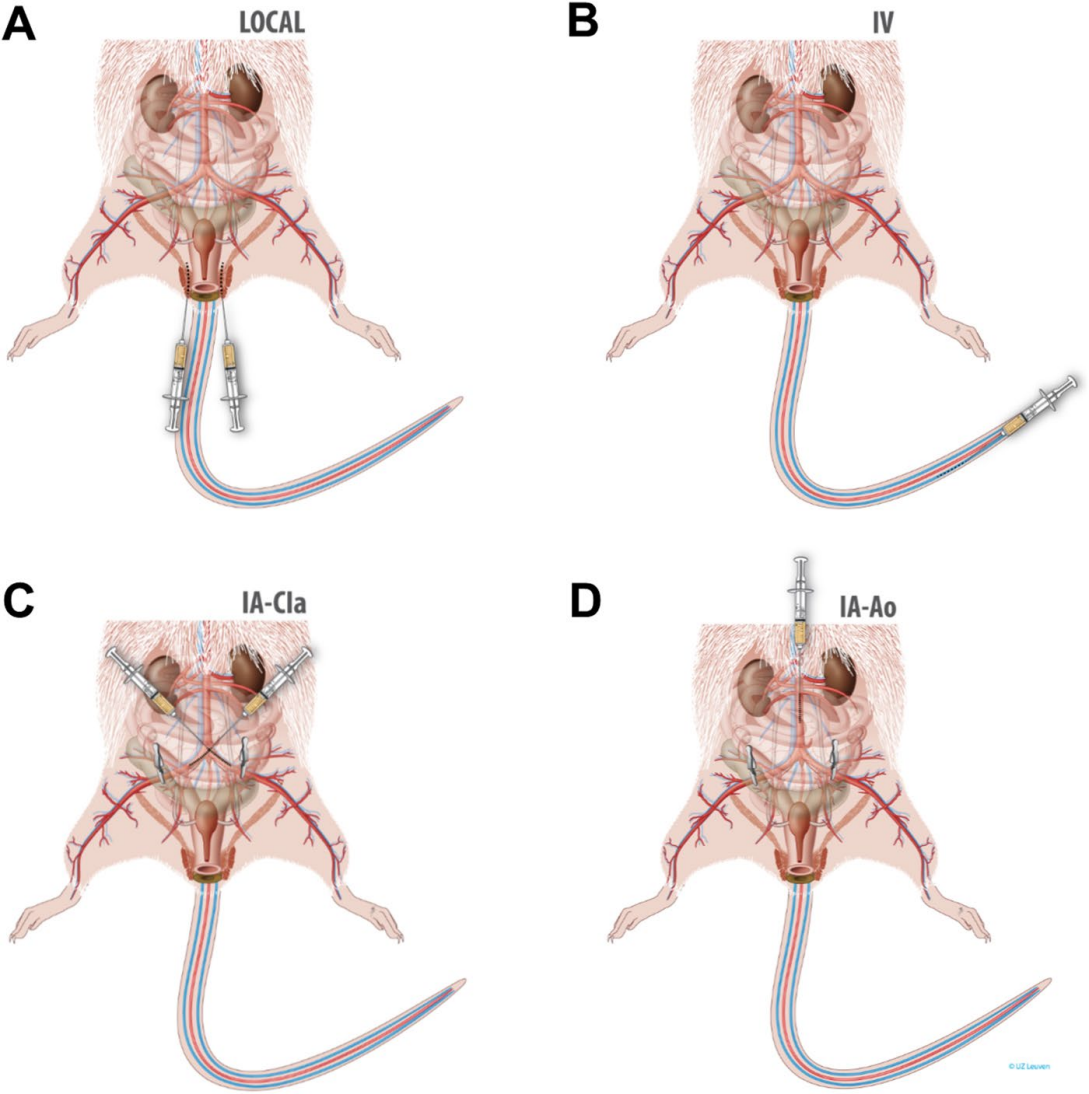
Routes of injection of rat mesoangioblasts (rMABS) for targeting the pelvicorgans in a simulated vaginal birth injury model in rat. (**A**) Local– injections were done laterally to the vagina. (**B**) Intravenous injection was performed in the tail vein. (**C**) Common Iliacs arteries- rMABs^eGFP/fLUC^ were injected in both common iliac arteries in two steps. The external iliacs were clamped and antegrade injection was done at a 45° angle along the vessel orientation. The procedure was performed bilaterally. (**D**) Aorta – rMABs^eGFP/fLUC^ were injected directly into the aorta 1.5 cm above the bifurcation. Before injection, both external iliac arteries were occluded with a vascular clamp.

Animals were kept under general anesthesia during administration of cells with 1.5% isoflurane in 100% oxygen at 1.5 L/min. Schematic drawings of all injection routes are displayed in Fig. 6. Local administration was done laterally at both sides of the vagina, using a 26 G vascular catheter needle (BD Neoflon Cannula, Becton Dickinson and company, Aalst, Belgium). IV injection was performed in the distal part of the tail vein using a 24 G vascular catheter (BD Insyte, Becton Dickinson and company). IA administrations were performed by either injecting in both the left and right common iliac arteries (IA-CIa), or by one injection in the aorta (IA-Ao). First a ventral midline laparotomy was performed. Next, for IA-CIa the right common iliac was dissected and a loose ligature was placed close to the aorta bifurcation to facilitate the insertion of the needle later on. In order to direct the flow of the injected cells towards the pelvicorgans, the right external iliac was occluded with a vascular clamp for 10–20 s. Antegrade catheterization at a 45°angle was performed using a 33 G needle (Acu-Needle, Acuderm, Fort Lauderdale-FL, USA) directly into the right common iliac artery. After injection, the needle was removed, and the injection site was compressed with a resorbable collagen membrane (Lyostypt®, B. Braun, Aesculap, Tuttlingen, Germany). The same procedure was performed on the contralateral side.

For the IA-Ao injection, the procedure was performed in a similar way, except that a single injection was administered after placing the loose ligature around the aorta 1.5 cm above the iliac bifurcation. After injection(s), the abdominal wall was closed with a 3-0 monofilament polypropylene suture in two layers (Prolene, Ethicon, Zaventem, Belgium). Prior to the formal experiments, we tested these routes with injections of Chinese ink in non-injured rats and tracked the carbon particles (n = 3 for each route). This was perfomed to determine the initial track of the injection. Immediately after injection, those animals were euthanized and pelvic organs were fixed in 4% of PFE for H&E staining.

### rMABs^eGFP/fLUC^ transplantation

One hour after simulated VD injury (n = 10/group) or sham (n = 5/group), rats were randomly assigned to receive 2 × 10^6^ rMABs^eGFP/fLUC^ either locally (perivaginal-bilateral), IV (tail vein), IA-CIa or IA-Ao. rMABs^eGFP/fLUC^ were resuspended in 30 µL (vagina) or 800 µL (IV or IA) of physiological solution. All rats received heparin (400 UI/kg; SC) 1 h before injection and the vasodilator isosorbide dinitrate (1 mg/kg; IV) 1 minute before injection.

### Distribution and viability of rMABs^eGFP/fLUC^*in vivo* and *ex vivo*

For *in vivo* bioluminescence (BLI), rats were first anesthetized with 1.5% of isoflurane in 100% of oxygen and then given a single injection containing d-luciferin potassium salt dissolved in phosphate-buffered saline (PBS) (126 mg/kg). None of the rats evaluated had the pelvic organ region shaved. Ten minutes after luciferin injection, the rats were placed in the imaging chamber (IVIS Spectrum, Perkin Elmer, X). Next, consecutive frames were acquired for 5 min until the maximum signal intensity was reached. A region of interest was drawn around the pelvic organs. The maximal radiance (p/s/cm2/sr) was measured within this region. Images were analyzed using Living Image version 4.5. BLI data was obtained 1 h, 1 day and 3 days following rMABseGFP/fLUC injection. *Ex vivo* BLI measurements were done at 3 or 7 days after injection. Rats were euthanized immediately after *in vivo* BLI and the urinary bladder, urethra, vagina, rectum, levator ani muscles, lungs and spleen were harvested and imaged similarly *ex vivo*. After BLI analysis, samples were snap frozen and stored in OCT at −80 °C until further analysis.

### Immunofluorescense

In order to confirm the presence of rMABs^eGFP/fLUC^ in the pelvicorgans, serial sections from the IA-Ao group (n = 2) were fixed with 4% PFA, permeabilized, and immunostained for eGFP (ab5450, Abcam,) and laminin (Ab11575; Abcam). Alexa 488 (Thermo Fisher Scientific) and Alexa 647 (Thermo Fisher Scientific) were used as secondary staining Ig G and Hoechst for nuclear staining. Isotype control antibodies were used as negative staining. Images were taken with a fluorescence microscope (ECLIPSE Ti, Nikon) using NIS elements software (Nikon).

### RT-PCR for GFP

RNA was extracted from frozen tissues following TRIzol extraction protocol (Life Technologies). 500 ng of RNA were retrotranscribed into cDNA (SSIII cDNA production kit, Thermo Fisher Scientific). qPCR was performed on 1/10 diluted cDNA, using ViiA7 384-plate reader (Thermo Fisher Scientific). Final primers concentration 100 nM, final reaction volume 10 µl, PGK and GAPDH as internal reference; thermal profile 95 °C 15 seconds, 60 °C 60 seconds, 40x. Primers are listed in Table 2. Data are shown as relative expression normalized on respective organ of local injections.

**Table 2:** List of primers used.

Genes	Forward	Reverse
PGK	ATGCAAAGACTGGCCAAGCTAC	AGCCACAGCCTCAGCATATTTC
GAPDH	CAACTCCCTCAAGATTGTCAGCAA	GGCATGGACTGTGGTCATGA
eGFP	CATGGTCCTGCTGGAGTTCGTG	CGTCGCCGTCCAGCTCGACCAG

### Statistics

Data are presented as mean ± SEM. ANOVA statistical tests were performed with Tukey post hoc tests if the samples were normally distributed. When not normally distributed, Kruskal-Wallis statistical tests were performed with Dunn’s test post hoc test. Intra-group comparison of viable cells at three different time points were analyzed by paired t-test. For gene expression analyses, 2-way ANOVA was applied to patterns measured over time. Significance was set at p < 0.05. Data were processed using GraphPad Prism version 5.00 for Windows (Graph Pad Prism, La Jolla, CA, USA).

## Electronic supplementary material

Supplementary Figures
