# Supplementary material for: Fate of mesoangioblasts in a vaginal birth injury model: influence of the route of administration
Source: Sci Rep. 2018 Jul 13;8:10604. doi: 10.1038/s41598-018-28967-w (PMC6045600; doi:10.1038/s41598-018-28967-w)
Supplement: Supplementary file 1 — Supplementary Figures [file 41598_2018_28967_MOESM1_ESM.docx]

**Fate of mesoangioblasts in a vaginal birth injury model: influence of the route of administration**

Marina Gabriela Monteiro Carvalho Mori da Cunha^1,2^; Giorgia Giacomazzi^3^; Geertje Callewaert^1, 2, 4^; Lucie Hympanova^1, 2, 5^; Francesca Russo^1,2^; Greetje Vande Velde^6^; Rik Gijsbers^7^; Maarten Albersen^8^; Maurilio Sampaolesi^3^; Jan Deprest^1, 2, 4*^

1 Centre for Surgical Technologies, Group Biomedical Sciences, KU Leuven, Leuven, Belgium

2 Department of Development and Regeneration, Woman and Child, Group Biomedical Sciences, KU Leuven, Leuven, Belgium

3 Translational Cardiomyology Lab, Stem Cell Biology and Embryology Unit, Department Development and Regeneration, KU Leuven, Leuven, Belgium

4 Pelvic Floor Unit, University Hospitals KU Leuven, Leuven, Belgium

5Institute for the Care of the Mother and Child, Third Faculty of Medicine, Charles University, Prague, Czech Republic

6 Molecular Small Animal Imaging Center, KU Leuven, 3000 Leuven, Belgium

7 Laboratory for Molecular Virology and Gene Therapy, KU Leuven, Flanders, Belgium

8 Department of Urology, University Hospitals Leuven, Leuven, Belgium

*corresponding author: jan.deprest@uzleuven.be


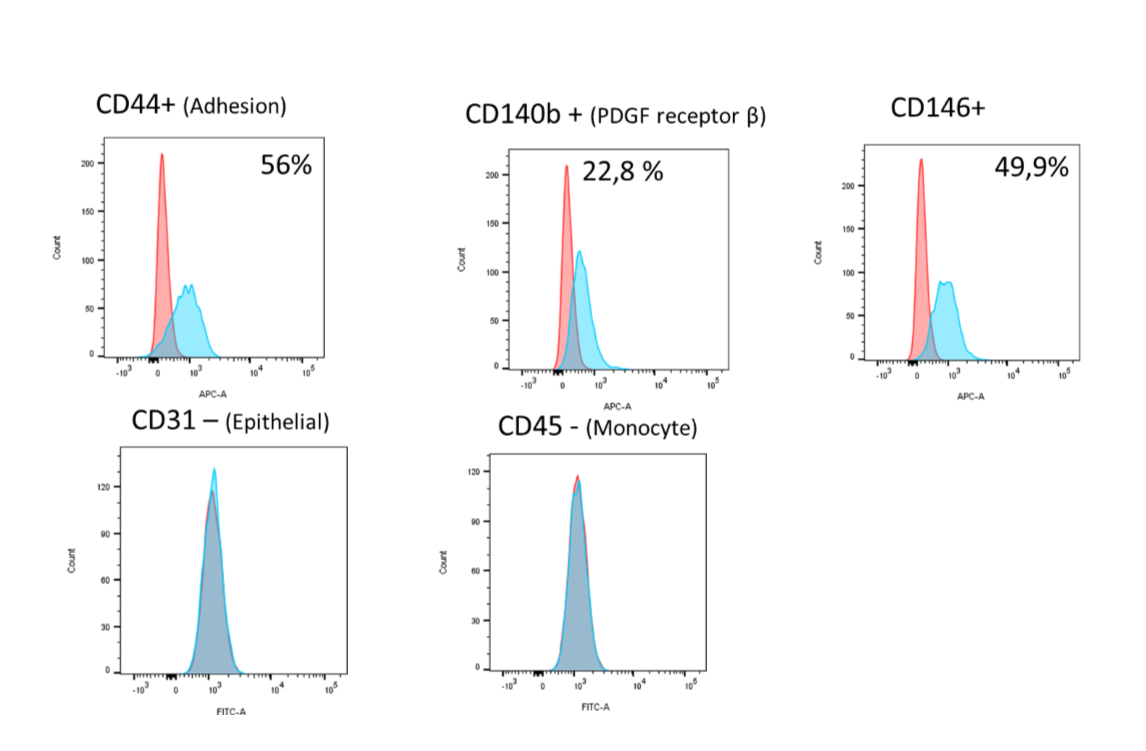


Supplementary figure 1: **characterization of MABS profile with flow cytometry analysis** Flow cytometry analysis on the isolated MABS (AP positive population, before viral transduction) show that rMABs are highly positive for MABs marker CD44, CD140b and CD146, while they are negative for CD31 and CD45.


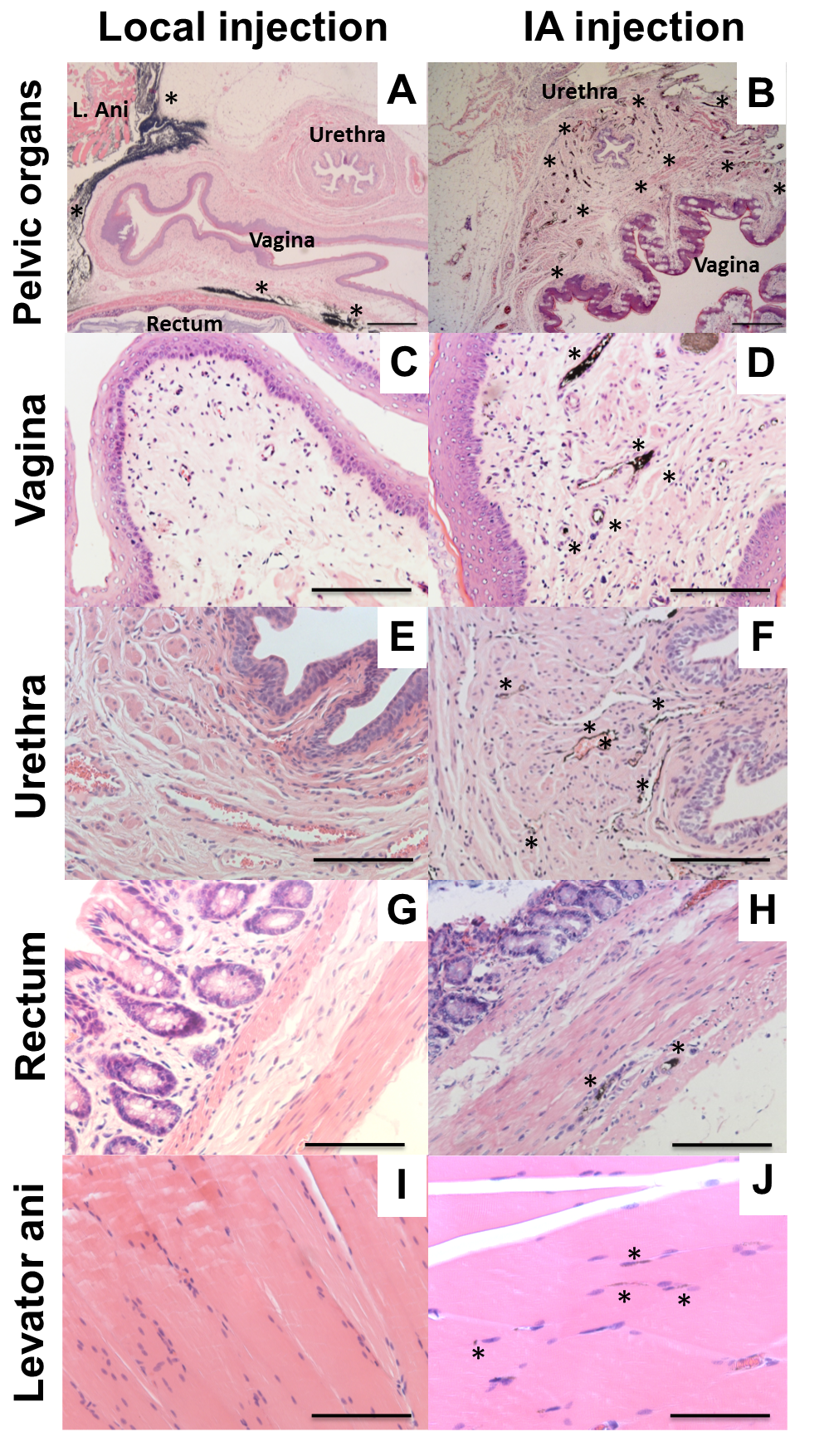


**Supplementary figure 2: Black carbon particles (Chinese ink) tracking after local and intra-arterial administration (H&E, A and B: 50X; C-J: 200X).** Black particles could be observed on histological sections in the connective tissue around the vagina, rectum and levator ani (A). There were no carbon particles visible within the interstitial and vascular spaces of these pelvic organs (C, E, G, I). (B). Black particles were homogeneously distributed in the pelvic organs when injection was performed by arterial route (B). Chinese ink could be observed in the interstitial and vascular space of these pelvic organs (D, F, H, J).


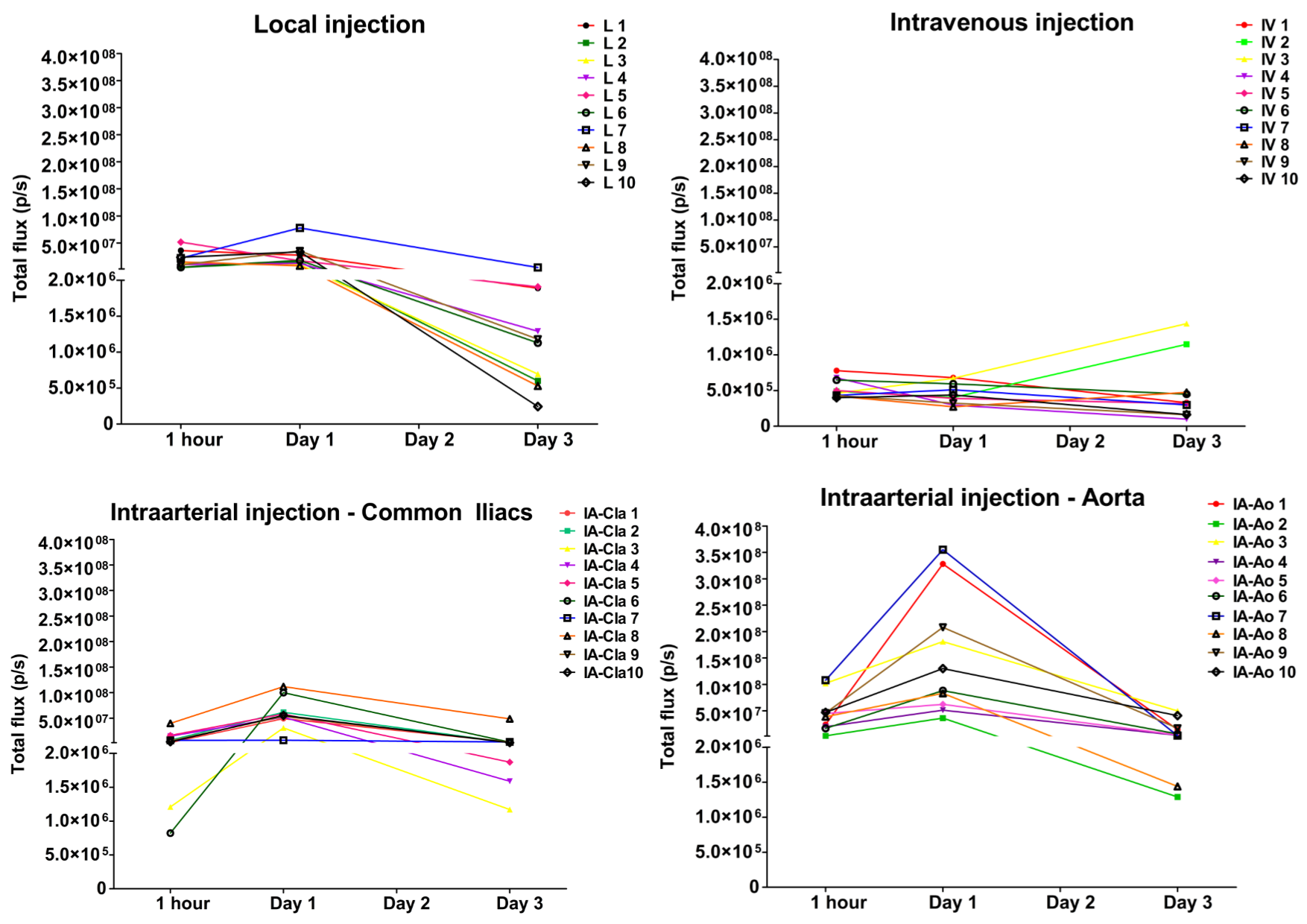


**Supplementary figure 3:** Individual data of rMABs^eGFP/fLUC^ presence in the pelvic floor area of rats submitted to simulated birth injury and treated with rMABs^eGFP/fLUC^  by local, intravenous and intra-arterial (common iliacs – IA-CIa or aorta- IA-Ao) routes.


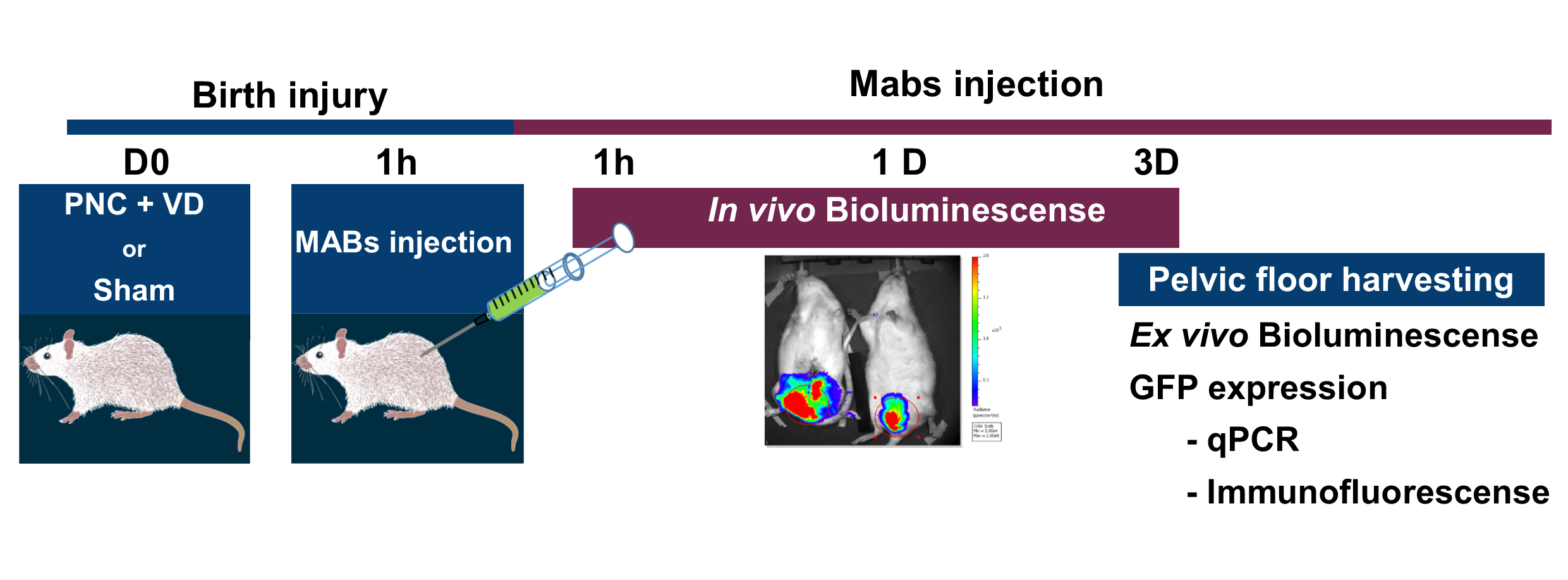


**Supplementary figure 4:** Time line of the experiment
